# Racial/ethnic and geographic differences in polybrominated diphenyl ether (PBDE) levels across maternal, placental, and fetal tissues during mid-gestation

**DOI:** 10.1038/s41598-020-69067-y

**Published:** 2020-07-22

**Authors:** Julia R. Varshavsky, Saunak Sen, Joshua F. Robinson, Sabrina Crispo Smith, Julie Frankenfield, Yunzhu Wang, Greg Yeh, June-Soo Park, Susan J. Fisher, Tracey J. Woodruff

**Affiliations:** 1 Program on Reproductive Health and the Environment, Department of Obstetrics, Gynecology and Reproductive Sciences, University of California, San Francisco, Mailstop 0132, 550 16th Street, 7th Floor, San Francisco, CA 94143 USA; 2 Department of Preventive Medicine, University of Tennessee Health Science Center, 66 North Pauline St, Memphis, TN 38163 USA; 3 Center for Reproductive Sciences and Department of Obstetrics, Gynecology and Reproductive Sciences, University of California, San Francisco, 513 Parnassus Avenue, San Francisco, CA 94143 USA; 4 Environmental Chemistry Laboratory, Department of Toxic Substances Control, California Environmental Protection Agency, 700 Heinz Ave # 200, Berkeley, CA 94710 USA

**Keywords:** Cell biology, Computational biology and bioinformatics, Developmental biology, Immunology, Microbiology, Molecular biology, Physiology, Stem cells, Systems biology

## Abstract

Prenatal polybrominated diphenyl ether (PBDE) exposures are a public health concern due to their persistence and potential for reproductive and developmental harm. However, we have little information about the extent of fetal exposures during critical developmental periods and the variation in exposures for groups that may be more highly exposed, such as communities of color and lower socioeconomic status (SES). To characterize maternal–fetal PBDE exposures among potentially vulnerable groups, PBDE levels were examined in the largest sample of matched maternal serum, placenta, and fetal liver tissues during mid-gestation among a geographically, racially/ethnically, and socially diverse population of pregnant women from Northern California and the Central Valley (*n* = 180; 2014–16). Maternal–fetal PBDE levels were compared to population characteristics using censored Kendall’s tau correlation and linear regression. PBDEs were commonly detected in all biomatrices. Before lipid adjustment, wet-weight levels of all four PBDE congeners were highest in the fetal liver (*p* < 0.001), whereas median PBDE levels were significantly higher in maternal serum than in the fetal liver or placenta after lipid-adjustment (*p* < 0.001). We also found evidence of racial/ethnic disparities in PBDE exposures (Non-Hispanic Black > Latina/Hispanic > Non-Hispanic White > Asian/Pacific Islander/Other; *p* < 0.01), with higher levels of BDE-100 and BDE-153 among non-Hispanic Black women compared to the referent group (Latina/Hispanic women). In addition, participants living in Fresno/South Central Valley had 34% (95% CI: − 2.4 to 84%, *p* = 0.07) higher wet-weight levels of BDE-47 than residents living in the San Francisco Bay Area. PBDEs are widely detected and differentially distributed in maternal–fetal compartments. Non-Hispanic Black pregnant women and women from Southern Central Valley geographical populations may be more highly exposed to PBDEs. Further research is needed to identify sources that may be contributing to differential exposures and associated health risks among these vulnerable populations.

## Introduction

Polybrominated diphenyl ethers (PBDEs) are a public health concern for pregnant women worldwide due to their developmental health impacts and widespread use as flame retardant chemicals in consumer and industrial products, including furniture, electronics, building materials, and child care articles^1^. PBDEs are biologically active endocrine disrupting compounds that can interfere with endogenous hormone activity, immune system function, and fetal brain development during pregnancy^2–4^. For 30 years, California had the strictest flammability standard in the world, with CA’s Technical Bulletin 117 (TB117) requiring the use of polyurethane foam in upholstered furniture and children’s products that was able to withstand an open flame for up to 12 seconds^5^. To comply with TB117, a pentaBDE mixture (BDE-47, -99, -100, -153, and -154) was marketed specifically for use in California, which drove heavy use of the commercial mixture in the United States and Canada between the 1970s and mid-2000s^1,5,6^.

Consequently, North Americans typically have the highest pentaBDE body burden levels worldwide, with some of the highest levels ever reported among pregnant women and new mothers in California^7,8^. PBDEs are loosely bound to foam and other treated products and can thus migrate easily into indoor air and dust, which is the primary exposure source for most pentaBDEs (i.e., through dust ingestion)^9–13^. They also bioaccumulate up the food chain and can be ingested through the dietary exposure pathway^1^. While PBDE levels have decreased in the U.S. population as newer furniture and consumer products have replaced older products over the last decade, legacy exposures remain due to their heightened environmental and biological persistence^14,15^. Moreover, the U.S. phase-out of pentaBDEs in the mid-2000s may be contributing to exposure disparities among lower socioeconomic (SES) populations in the United States (e.g., from continued pentaBDE exposures in low-income households with more second-hand purchases and/or deteriorating furniture)^10,14–20^.

PBDEs are present in human placental or fetal tissues and may not be readily metabolized during pregnancy^21–23^, which can potentially enhance susceptibility to developmental perturbations and adverse health outcomes. These brominated flame retardants are thought to adversely influence pregnancy and child health outcomes by disrupting thyroid hormone (TH) homeostasis and developmental processes dependent on TH^24^. PBDEs may also directly induce toxicity in cells by eliciting oxidative stress, DNA damage, mitochondrial dysfunction, apoptosis, and altering neurotransmitter signaling pathways^3^. Perinatal and in utero exposures have been associated with adverse birth outcomes^25^, maternal health complications (e.g., preeclampsia^2^), and neurodevelopmental impacts in children (e.g., reduced cognitive ability and executive functioning)^3,26,27^. A rigorous systematic review and meta-analysis found sufficient evidence for a positive association between in utero PBDE exposures and reduced intelligence quotient (but not attention-related disorders) among U.S. children^27^.

Furthermore, biomonitoring studies have shown the fetus may be more highly exposed to PBDEs^21,28–30^, possibly due to their lipophilic properties (making them more likely to transfer to fatty tissue) or their structural similarities with THs, which are readily transferred from mother to fetus during pregnancy in order to facilitate fetal brain development^24,28^. Moreover, the capacity to metabolize PBDEs via the cytochrome P450 pathway is less mature in the fetal liver, which may contribute to PBDE accumulation in fetal tissues during pregnancy^21^. An additional critical factor in maternal–fetal development and PBDE transfer during pregnancy is the placenta, which can itself be a direct target for PBDE toxicity^22^ and can also influence the transport and exposure of PBDEs to the fetus^28^. Consequently, because of differential maternal–fetal PBDE exposures and their developmental health effects, it is important to understand the relationship between maternal, placental, and fetal exposures during the critical period of placentation and fetal development that occurs in the second trimester of pregnancy. Moreover, levels of these persistent chemicals continue to accumulate in young infants and children (e.g., through early life exposures to breast milk and dust), increasing concerns about potential cumulative health impacts as children mature^8,31–33^.

While previous research has characterized PBDEs in maternal and fetal tissues during pregnancy^14,21,28–30,34^, several shortcomings have restricted interpretation with regard to fetal exposures during sensitive periods of development. First, many studies have limited sample size (i.e., access to paired maternal–fetal samples), ranging from *n* = 72^30^ to *n* = 80 paired maternal–fetal samples (*n* = 50 matched maternal serum, placental, and fetal liver samples)^21^, and generalizability (i.e., studies conducted in uniquely exposed populations outside the United States, such as pregnant electronic workers in China)^30,34^. Second, the majority of studies have focused on maternal and cord serum rather than other fetal or placental tissues^14,29^. In addition, the timing of sample collection has been largely constrained to labor and delivery^14,28,29^ rather than earlier in gestation (when transplacental transfer of maternal thyroid hormone is greatest^35^), which would be more relevant for assessing fetal PBDE exposures during critical prenatal windows of vulnerability.

Therefore, we measured PBDE concentrations in a larger sample of matched maternal–fetal tissues (maternal serum, placenta, and the fetal liver; *n* = 180) during mid-gestation in order to examine the correlation of PBDE congeners across the maternal–fetal interface among a diverse pregnancy population in California. We sought to investigate whether maternal levels can predict fetal levels, thereby assessing the utility of maternal serum as a surrogate for fetal exposure assessment, and to determine the exposure relationship between maternal and fetal compartments in order to gain a better understanding of the extent to which the fetus may be at higher (or lower) risk because of higher (or lower) exposures based on maternal levels. Additional socio-demographic factors which may influence maternal–fetal PBDE exposures (*i.e.*, race/ethnicity and geography) were also examined.

## Methods

### Study recruitment and sample collection

**Figure 1:**
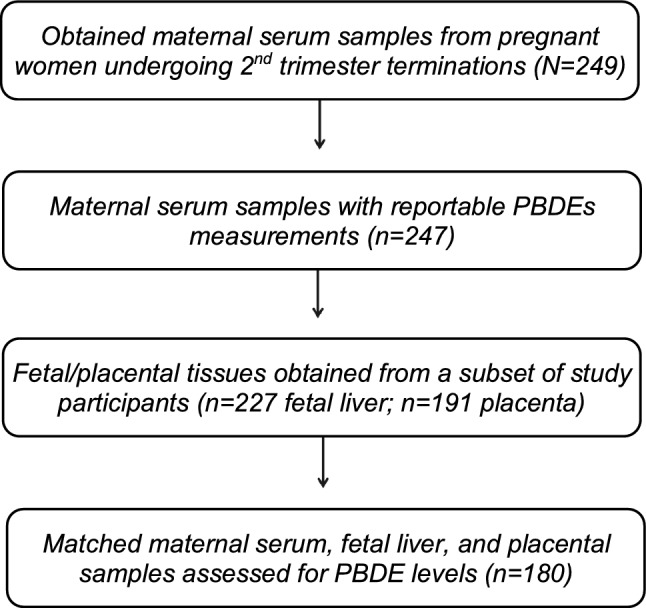
Participant recruitment and biological samples collected from pregnant women during mid-gestation at the Women’s Options Center (WOC) in Northern California from 2008–16. We obtained maternal serum samples and questionnaire data from 249 pregnant women undergoing elective terminations during mid-gestation in four study waves from 2008–16: wave 1 (2008–09; *n* = 25), wave 2 (2011–12; *n* = 36), wave 3 (2014; *n* = 50), and wave 4 (2014–16; *n* = 138). Polybrominated diphenyl ether (PBDE) levels were measured in 247 maternal serum samples. Fetal liver (study waves 2–4; 2014–16; *n* = 227) and placental tissues (study waves 3–4; 2014–16; *n* = 191) were also collected in a subset of women. After excluding twins, PBDE measurements were obtained for 180 matched samples of maternal serum, fetal liver, and the placenta in two study waves from 2014–16.

We recruited 249 pregnant women receiving an elective termination during mid-gestation at the Women’s Options Center (WOC) in Northern California across four study waves from 2008–16: wave 1 (*n* = 25; 2008–09), wave 2 (*n* = 36; 2011–12), wave 3 (*n* = 50; 2014), and wave 4 (*n* = 138; 2014–16) (Fig. 1). We have previously published data that were based on study waves 2 and 3 which cover the years 2011–14^21^, and here we add further data from years 2014–16 (study wave 4) to give a more robust picture of PBDE levels across maternal–fetal tissues with a larger sample size. Maternal serum samples were obtained from each participant during the procedure visit, and questionnaires were administered in order to ascertain population characteristics including educational attainment, employment status, insurance type, marital status, race/ethnicity, zip code, and smoking status (with study participation restricted to non-smokers in the first and fourth study waves, although current and former smokers were allowed to participate in the second and third study waves, respectively^15^). Biological characteristics, such as weight and gestational age, were measured at the clinical visit and subsequently abstracted from each participant’s medical chart. When possible, fetal sex was determined through gonad examination following the medical procedure. PBDE levels were later measured in 247 maternal serum samples. Fetal liver and placental tissues were collected from a subset of participants in WOC study waves 2–4 (*n* = 227; 2011–16) and study waves 3–4 (*n* = 191; 2014–2016), respectively. Non-matched samples and twins were removed, leaving a total of *n* = 180 matched maternal serum, placental, and fetal liver PBDE measurements available for statistical analysis (Fig. 1). All study protocols were approved by the University of California, San Francisco (UCSF) institutional review board prior to recruitment; written and verbal consent were obtained from each study participant at the time of enrollment.

### Chemical analysis

Biological samples were collected at UCSF, with whole blood samples collected in red top Vacutainer tubes and centrifuged to separate the serum at 3,000 RPM for 10 min at 4 degrees Celsius and serum further aliquoted into pre-screened sterilized amber vials using glass pipettes and stored at − 80 ∘C until further analysis. Serum and tissue samples were then transferred to the Environmental Chemistry Laboratory at the Department of Toxic Substances and Control (DTSC) in Berkeley, California for chemical analysis. Nineteen total PBDE congeners (BDE-17, -28, -47, -66, -85, -99, -100, -153, -154, -183, -196, -197, -201, -202, -203, -206, -207, -208, and -209) were analyzed in each biological tissue according to tissue-specific protocols (Table S1)*.* Details of the serum extraction method have been published elsewhere^9,29^. Briefly, thawed serum samples were spiked with carbon-labeled internal standards and denatured with formic acid. The PBDE congeners were extracted from serum using Oasis HLB cartridges (3 cc, 500 mg, Waters Corp.; Milford, MA) and an automated sample extraction system (RapidTrace, Biotage; Uppsala Sweden). Sample extracts then underwent cleanup using acidified silica (500 °C prebaked, manually packed, 3 cc). As described in detail elsewhere^15,21^, measured values from National Institute of Standards and Technology Standard Reference Material No. 1958 (NIST SRM) samples were similar to certified PBDE values, ranging from 102 ± 4% (BDE-99) to 119 ± 7% (BDE-100). PBDE concentrations within samples were corrected using labelled surrogates (isotope dilution), with average recoveries ranging from 93 ± 14% (for BDE-28L) to 111 ± 20% (BDE-99L). Total cholesterol and triglycerides were measured enzymatically by Boston Children’s Hospital (Boston, MA) and subsequently used to calculate total serum lipid levels for each study participant using the Phillips formula^36^.

Fetal liver and placental samples were analyzed using our liver analytical method with slight modification^21^. Before sample extraction, only placenta samples were lyophilized. Briefly, the samples were homogenized and spiked with carbon-labeled internal standards. The samples were then denatured with hydrochloric acid and extracted with 1:1 hexane:methyl *tert*-butyl ether (MTBE). Aqueous potassium chloride solution was added to remove potentially co-extracted aqueous compounds and the organic layer was re-extracted. The extract was dried in a pre-weighed, pre-baked aluminum weighing dish to determine lipid content via gravimetric analysis. After lipid determination, the samples were reconstituted in hexane and the lipids removed from the sample using concentrated sulfuric acid, followed by a cleanup with acidified silica (500 °C prebaked, manually packed, 3 cc) using the automated SPE system (RapidTrace, Biotage; Uppsala Sweden). All samples were analyzed on a DFS magnetic sector GC-HRMS system (Thermo Scientific, Bremen, Germany). Isotopically-labeled internal surrogate mix standards (IS) were used for quantitation (Wellington Laboratories, Inc., Guelph, Ontario, Canada). As previously described^21^, certified reference materials were not readily available for PBDEs in liver or placental matrices at the time of sample processing, so NIST SRM 1958 (human serum) were processed with liver and placenta samples to determine average recoveries of the extraction process^21^. Briefly, measured values from SRM samples were within reasonable error ranges of certified PBDEs (BDE-28: 105 ± 11% (liver) 99 ± 11% (placenta), BDE-47: 110 ± 7% (liver) 103 ± 14% (placenta), BDE-99: 94 ± 5% (liver) 93 ± 7% (placenta), BDE-100: 94 ± 6% (liver) 96 ± 5% (placenta), and BDE-153: 105 ± 8% (liver) 101 ± 9% (placenta)). PBDE concentrations in liver and placenta samples were also corrected using labelled surrogates, with average recoveries ranging from 44 ± 13% (BDE-28L) to 58 ± 21% (BDE-99L).

### Statistical analysis

Initially, we computed detection frequencies and examined univariate statistics for all 19 PBDE congeners in each biological matrix (with wet-weight units expressed as ng/ml in maternal serum and ng/g in the placenta and fetal liver) (Table S1). Lipid-adjusted PBDE levels (expressed as ng/g lipid in all tissues) were also calculated by dividing wet-weight PBDE values by tissue-specific total lipid levels (expressed as mg/ml in maternal serum and mg/g in the placenta and fetal liver). Five PBDE congeners were selected for further statistical analyses based on > 50% detection frequency (BDE-47, -99, -100, and -153 in all biological tissues; BDE-28 in placenta and fetal liver only)^37^.

All correlation and regression analyses involving PBDE concentrations were performed using censored approaches for analyzing left-censored chemical data (i.e., non-zero PBDE values below the laboratory method detection limit, or MDL). Censored Kendall’s tau correlation was used as a non-parametric approach to estimate the rank order (monotonic) correlation between two left-censored variables (PBDE congeners within and between maternal–fetal tissues)^38^. Summary statistics and linear regression associations for PBDE congeners were computed using censored maximum likelihood estimation (MLE), a parametric approach which accounts for multiple MDLs and detection frequencies^37^. We assumed a log-normal distribution and modeled PBDE levels as outcome (dependent) variables of interest in bivariate regression (ANOVA) and multivariable MLE regression models. If one of the predictor variables was also censored, regular linear regression was used instead because censored multivariable methods typically do not account for left-censored data in both the exposure and outcome distributions. In that case, PBDE levels were log-transformed prior to analysis (to approximate a normal distribution) and < MDL values were substituted with MDL/sqrt2. Covariates were considered for inclusion in multivariable models based on a priori literature and whether they were associated with the exposure and outcome and/or whether they changed effect estimates on exposure-outcome associations by more than 10%. All statistical analyses were performed in R (Version 3.5.1). We reported the 95% confidence interval (95% CI) for regression results and *p*-values for ANOVA tests, defining significance as *p* < 0.05 and marginal significance as *p* < 0.10.

We calculated MLE estimates of the median, interquartile range (IQR: 25th–75th percentile), minimum, and maximum values based on a log-normal distribution for all five PBDE congeners (Table S1). Summary statistics were also calculated for the sum of four congeners (ΣPBDE4 = BDE-47, -99, -100, and -153) and the sum of five congeners (ΣPBDE5 = BDE-28, -47, -99, -100, and -153). PBDE levels were compared between maternal and fetal/placental tissues using regression models as described and by directly calculating the ratio of each congener in matched samples of fetal liver:maternal serum, fetal liver:placenta, and placenta:maternal serum. Initially, PBDE exposure differences across maternal, placental, and fetal tissues were tested without accounting for matched biological samples (i.e., comparing median maternal–fetal PBDE exposures and assuming independence between groups). We then accounted for matched within-person samples of maternal serum, placental, and fetal liver tissues (i.e., comparing individual-level maternal–fetal PBDE exposures assuming dependence and using person as the pairing factor). This approach required the use of censored intervals rather than MDLs^37^. The ratio of PBDE levels between paired maternal–fetal tissues was calculated by computing PBDE level cross-tissue differences on the natural log scale. To further investigate the relationship between maternal and fetal PBDE exposures during mid-gestation, maternal serum (exposure) and fetal liver (outcome) PBDE levels were modeled as the main exposure and outcome variables of interest (both natural log-transformed). Additional explanatory covariates included gestational age, fetal sex, study wave, and total serum lipid levels^21,23,39^. This analysis was restricted to paired PBDE measurements in fetal liver and maternal serum collected during study waves 2–4 (2011–16; *n* = 224).

Median PBDE levels were then compared across categorical subgroups for each of the following population characteristics (in bivariate MLE models): Maternal age, body mass index (BMI), birth country, educational attainment, employment status, gestational age, insurance type, parity, race/ethnicity, geographical region of residence, fetal sex, and sample collection year (Table 1). We further examined racial/ethnic and geographic differences in maternal–fetal PBDE levels using multivariable MLE regression, where race/ethnicity and region of residence were each modeled separately as main independent variables (exposures) of interest. We categorized race/ethnicity, which refers to both the social (rather than biological) construct of race and the cultural groups with which someone identifies (e.g., Latina)^40–44^, into three mutually exclusive subgroups as follows: Latina/Hispanic, Non-Hispanic Black, Non-Hispanic White, and Other (including Asian/Pacific Islander and to a lesser extent Native American). Geographical regions of residence were divided into three subgroups based on residential county zip code as follows: (1) SF Bay Area, including Alameda, Contra Costa, Marin, Napa, San Francisco, San Mateo, Santa Clara, Solano, and Sonoma Counties; (2) Fresno/South Central Valley, including Fresno, Kings, Kern, South San Joaquin, Madera, and Tulare Counties; and (3) North Central Valley, including San Joaquin, Stanislaus, Merced, and Monterey Counties in addition to the Coast, Sierras, Nevada, and Sacramento counties (including Shasta, Tehama, Glenn, Butte, and Colusa, as well as El Dorado, Solano, Sutter, Yuba, Yolo, and Placer Counties). The percent (%) difference (95% CI) in wet-weight PBDE levels between racial/ethnic (or geographic) subgroups was calculated from congener-specific and biomatrix-specific multivariable MLE regression models, assuming a log-normal PBDE distribution and adjusting for race/ethnicity (geographic model), region of residence (race/ethnicity model), educational attainment, sample collection year, gestational weeks, and tissue-specific total lipid levels as independent (explanatory) covariates. Although women born outside of the United States have been shown in previous studies (and in our data) to have lower PBDE exposures than US-born women^14,45^, we did not include birth country status in multivariable models because the stratified sample sizes were small (i.e., there were no foreign-born white or black women in our study population). Instead, we assessed the influence of birth country on PBDE levels by restricting the multivariable analysis to US-born only participants (*n* = 105). Latina/Hispanic participants and SF Bay Area residents were used as referent groups in MLE regression models because they comprised the largest racial/ethnic and geographic subgroups in the study population.

**Table 1:** Population characteristics with unadjusted geometric mean (GM) and 95% confidence interval (95% CI) of wet-weight BDE-47 levels in maternal, placental, and fetal liver tissues among pregnant women during mid-gestation at the Women's Options Center (WOC) in Northern California (study waves 3–4; 2014–16; n = 180)

Characteristic	*n* (%)	Fetal liver BDE-47 (ng/g)	p-value	BDE-47 (ng/g)	p-value	Maternal serum BDE-47 (ng/ml)	p-value
**Maternal age**							
< 20 years	27 (15)	0.32 (0.23, 0.44)	0.764	0.12 (0.09, 0.15)	0.81	0.17 (0.13, 0.22)	0.329
20–24 years	74 (41)	0.29 (0.20, 0.43)		0.12 (0.09, 0.15)		0.20 (0.14, 0.27)	
25–29 years	42 (23)	0.27 (0.18, 0.42)		0.11 (0.08, 0.15)		0.18 (0.13, 0.25)	
≥ 30 years	37 (21)	0.30 (0.19, 0.47)		0.11 (0.08, 0.15)		0.16 (0.11, 0.23)	
**Body mass index (BMI)**							
Normal/under (< 25 kg/m^2^)	79 (44)	0.27 (0.22, 0.32)	0.310	0.11 (0.09, 0.13)	0.697	0.17 (0.15, 0.20)	0.834
Overweight (25–30 kg/m^2^)	52 (29)	0.29 (0.22, 0.40)		0.12 (0.10, 0.15)		0.18 (0.14, 0.23)	
Obese (≥ 30 kg/m^2^)	49 (27)	0.34 (0.25, 0.46)		0.11 (0.09, 0.14)		0.19 (0.15, 0.24)	
**Birth country**							
U.S. born	105 (58)	0.28 (0.24, 0.33)	0.045*	0.13 (0.11, 0.14)	0.014*	0.18 (0.16, 0.20)	0.055
Foreign born	16 (9)	0.15 (0.10, 0.25)		0.08 (0.06, 0.11)		0.12 (0.08, 0.16)	
**Education**							
≤ High school	86 (48)	0.32 (0.27, 0.39)	0.423	0.12 (0.11, 0.14)	0.298	0.21 (0.18, 0.24)	0.022*
≥ Some college	93 (52)	0.27 (0.21, 0.35)		0.11 (0.09, 0.13)		0.16 (0.13, 0.19)	
**Employment**							
Employed	70 (39)	0.26 (0.21, 0.32)	0.855	0.10 (0.09, 0.12)	0.936	0.18 (0.15, 0.21)	0.559
Unemployed	73 (41)	0.28 (0.21, 0.38)		0.10 (0.08, 0.12)		0.20 (0.16, 0.25)	
**Gestational age**							
< 19 weeks	47 (26)	0.37 (0.29, 0.47)	0.102	0.12 (0.10, 0.14)	0.811	0.18 (0.15, 0.22)	0.989
≥ 19 weeks	133 (74)	0.27 (0.20, 0.36)		0.11 (0.09, 0.14)		0.18 (0.14, 0.23)	
**Insurance**							
Public	136 (76)	0.32 (0.28, 0.37)	0.011*	0.12 (0.11, 0.13)	0.037*	0.19 (0.17, 0.21)	0.509
Private/self-pay	39 (22)	0.20 (0.15, 0.27)		0.09 (0.07, 0.11)		0.16 (0.13, 0.20)	
**Parity**							
0 prior live births	73(41)	0.24 (0.20, 0.29)	0.055	0.10 (0.08, 0.11)	0.032*	0.15 (0.13, 0.17)	0.011*
≥ 1 prior live births	107 (59)	0.33 (0.26, 0.43)		0.13 (0.10, 0.15)		0.20 (0.17, 0.25)	
**Race/ethnicity**							
Latina/Hispanic	67 (37)	0.30 (0.24, 0.37)	0.063	0.12 (0.10, 0.14)	0.104	0.19 (0.16, 0.22)	0.021*
Non-Hispanic Black	47 (26)	0.28 (0.20, 0.39)		0.12 (0.09, 0.15)		0.18 (0.14, 0.24)	
Non-Hispanic White	40 (22)	0.36 (0.26, 0.51)		0.12 (0.09, 0.15)		0.20 (0.15, 0.26)	
Asian/Pacific Islander/Other	26 (14)	0.22 (0.15, 0.32)		0.09 (0.07, 0.12)		0.13 (0.09, 0.17)	
**Region of residence**							
SF Bay Area	99 (55)	0.26 (0.22, 0.30)	0.103	0.11 (0.09, 0.12)	0.395	0.17 (0.15, 0.19)	0.016*
S. Central Valley/Fresno	24 (13)	0.38 (0.26, 0.55)		0.13 (0.10, 0.17)		0.25 (0.19, 0.34)	
N. Central Valley/Coast/Sierras	39 (22)	0.31 (0.23, 0.43)		0.12 (0.09, 0.15)		0.17 (0.13, 0.21)	
**Fetal sex**							
Male	92 (51)	0.30 (0.25, 0.35)	0.978	0.11 (0.10, 0.13)	0.956	0.18 (0.16, 0.21)	0.997
Female	85 (47)	0.29 (0.22, 0.37)		0.11 (0.09, 0.13)		0.18 (0.15, 0.22)	
**Collection year**							
2014	81 (45)	0.39 (0.33, 0.47)	< 0.001**	0.13 (0.12, 0.15)	0.001**	0.17 (0.14, 0.20)	0.496
2015	91 (51)	0.22 (0.17, 0.29)		0.10 (0.08, 0.11)		0.19 (0.15, 0.23)	
2016	8 (6)	0.32 (0.18, 0.59)		0.16 (0.10, 0.26)		0.19 (0.12, 0.32)	

^†^Number missing = 56, 1, 4, 1, 23, 2, 5, for birth country, education, employment, insurance, race/ethnicity, and region of residence, respectively.

*p < 0.05; **p < 0.01.

### Ethics approval and consent to participate

This study was approved by the University of California, San Francisco Institutional Review Board (UCSF IRB). Written and verbal consent were obtained from study participants prior to sample collection in accordance with the Declaration of Helsinki.

### Consent for publication

Not applicable.

## Results

The majority of women in this study population were under 30 years old (75%) and in their second trimester of pregnancy (Table 1). About 60% of women had at least one prior live birth. Education level was evenly divided between ≤ High School and at least some college, while most women used public rather than private health insurance (76 compared to 22%, respectively). The study population was racially/ethnically diverse, with 37% of women identifying as Latina/Hispanic, 26% as Non-Hispanic Black, 22% as Non-Hispanic White, and 14% as Asian/Pacific Islander/Other, while race/ethnicity remained unknown/missing for 17.8% of study participants. The majority of women were born in the United States; however, birth country was missing for *n* = 56 (31%) study participants. Of note, a limited number of samples (*n* = 10; 7% of the sample set) were collected in 2016. Women born outside the United States had lower PBDE levels than US-born women, which is consistent with previous literature^14,45^. Higher educational attainment (post high school education compared to high school degree equivalency or less) was also associated with reduced serum PBDE levels. Additionally, shorter gestational age was modestly associated with higher BDE-47 levels in the fetal liver, while earlier collection years were associated with higher PBDE levels (Table 1).

We found that four PBDE congeners (BDE-47, -99, -100, and -153) were commonly detected in all three biomatrices (maternal, placental, and fetal tissues), while BDE-28 was widely detected in placental and fetal tissues only (with < 20% detection in maternal serum) (Table 2). BDE-47 was the most prevalent congener (≥ 99% detection frequency in all biological tissues), while BDE-99, -100, and -153 were detected in ≥ 72% of all samples. Method detection limits for all 19 congeners were an order of magnitude higher in maternal serum than in fetal/placental tissues, with BDE-209 having the highest MDLs (by ~ 1 to 2 orders of magnitude), relatively low detection frequencies (≤ 11%), and the highest number of non-reports (due to sample loss or surrogate failure) compared to other PBDE congeners in all three tissues (Table S1).

**Table 2:** Descriptive statistics^a^ of PBDE levels in matched samples of maternal serum, placenta, and fetal liver tissues during mid-gestation (study waves 3–4; 2014–16; n = 180)

Congener	MDL		% Detection	Wet weight (ng/ml)			Lipid adjusted (ng/g lipid)		
				Median	IQR	Max	Median	IQR	Max
**Fetal liver**									
BDE-28	0.008		66	0.02	0.02	0.2	0.9	0.9	7.0
BDE-47	0.042		98	0.20	0.3	4.1	11	12	164
BDE-99	0.018		96	0.06	0.08	2.3	3.4	3.3	79
BDE-100	0.009		95	0.05	0.06	0.7	2.4	2.5	25
BDE-153	0.016		88	0.10	0.1	0.8	5.1	5.5	31
ΣPBDE4^b^	–		–	0.60	0.7	7.3	30	26	252
ΣPBDE5^c^	–		–	0.70	0.8	7.4	34	29	255
BDE-28	0.006		64	0.009	0.006	0.05	1.0	0.7	3.9
BDE-47	0.017		100	0.09	0.08	0.9	9.5	8.5	107
BDE-99	0.009		99	0.03	0.02	0.4	3.0	2.2	46
BDE-100	0.004		100	0.02	0.02	0.1	1.9	1.8	16
BDE-153	0.009		92	0.04	0.04	0.2	4.2	3.8	21
ΣPBDE4^b^	–		–	0.20	0.2	1.3	23	18	152
ΣPBDE5^c^	–		–	0.20	0.2	1.3	26	20	156
**Maternal serum**									
BDE-28	0.020		16	0.02	0.01	0.06	2.9	1.6	10
BDE-47	0.044		98	0.20	0.20	1.9	22	22	265
BDE-99	0.026		85	0.06	0.04	0.7	8.0	5.9	85
BDE-100	0.017		78	0.04	0.04	0.3	5.9	6.2	43
BDE-153	0.031		72	0.08	0.07	0.4	12	10	55
ΣPBDE4^b^	–		–	0.50	0.40	2.7	67	51	371
ΣPBDE5^c^	–		–	–	–	–	–	–	–

*IQR* interquartile range (25th–75th percentile), *MDL* method detection limit.

^a^Summary statistics account for left-censored PBDE levels (below MDL) using maximum likelihood estimation.

^b^ΣPBDE4 includes congeners with > 50% detection frequency in all tissues (BDE-47, -99, -100, and -153).

^c^ΣPBDE5 includes congeners with > 50% detection frequency in placenta and fetal liver (BDE-28 + ΣPBDE4).

**Figure 2:**
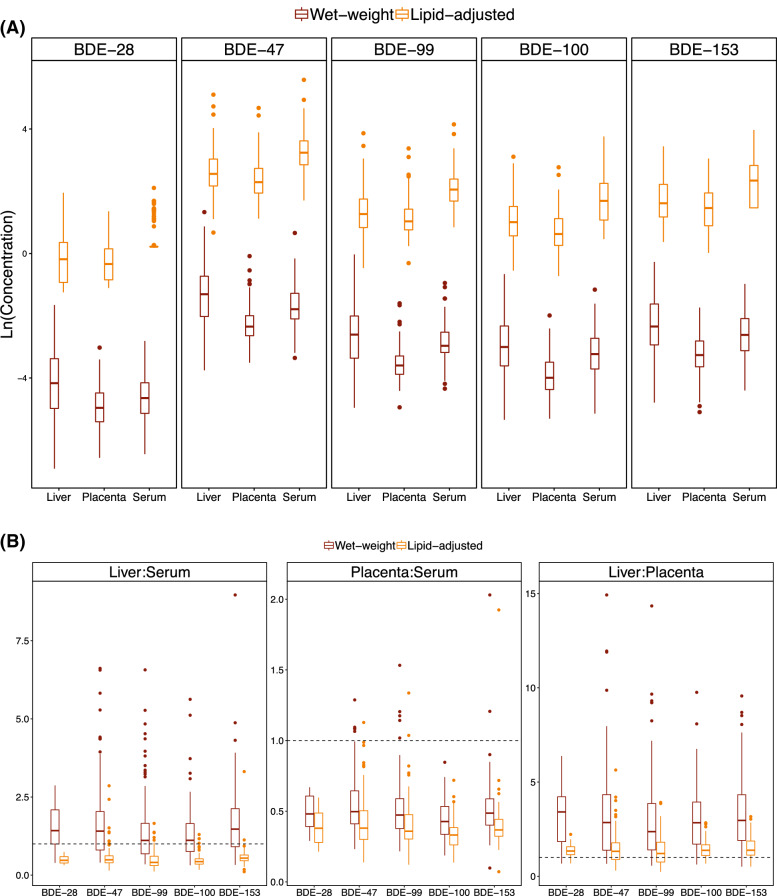
PBDE levels **(A)** and ratios **(B)** across maternal-placental-fetal biological matrices during mid-gestation (study waves 3 and 4; 2014–16; n = 180). Wet-weight units expressed as ng/g in fetal liver and placenta and ng/ml in maternal serum. Lipid-adjusted units expressed as ng/g lipid in all biological tissues. BDE-28 detected in only 16% of maternal serum samples. *P*-values < 0.05 in **(A)** from ANOVA test of group mean differences on log scale using censored maximum likelihood regression models. Dashed line represents PBDE level ratio equal to one between biological tissues.

The relationship of PBDE levels in maternal–fetal tissues varied based on whether or not wet-weight or lipid-normalized levels were examined. Wet-weight PBDE levels were highest in the fetal liver (~ 12 to 46% higher; *p* < 0.001), while lipid-adjusted concentrations were highest in maternal serum (~ 48 to 59% higher; *p* < 0.001) (Fig. 2A and Table 2). For all congeners, the ratio of PBDE levels between the fetal liver and maternal serum also varied by lipid-adjustment (> 1.0 prior to lipid-adjustment and < 1.0 after lipid-adjustment) (Fig. 2B and Table 3). In contrast, PBDE levels were consistently lowest in the placenta, regardless of lipid-adjustment, with maternal serum values for BDE-47 that were more than twice as high as placental values (minimum ratio of 0.14 and up to 4.1 times higher). Adjusted regression models of the association between maternal and fetal PBDE exposures indicated that maternal serum PBDE levels explained ~ 50% of the variability in fetal liver PBDE levels for all congeners except BDE-99, for which the adjusted R^2^ value was ~ 0.20 (Table S2. Additionally, wet-weight PBDE levels were more highly correlated with lipid concentrations in the fetal liver than in the placenta or maternal serum, while tissue-specific total lipid levels were higher and more variable in the fetal liver compared to the placenta (Table S3).

**Table 3:** Unadjusted percent (%) difference (95% CI)^a^ in PBDE levels between maternal and fetal/placental tissues during mid-gestation (study waves 3–4; 2014–16; n = 180)

Congener	Wet-weight (ng/ml, ng/g)		Lipid-adjusted (ng/g lipid)	
	% Difference (95% CI)	p-value	% Difference (95% CI)	p-value
Biomatrix				
**BDE-47**				
Maternal serum	Referent	< 0.001	Referent	< 0.001
Fetal liver	46 (24, 73)*		− 48 (− 55, − 39)*	
Placenta	− 42 (− 51, − 32)*		− 58 (− 64, − 51)*	
**BDE-99**				
Maternal serum	Referent	< 0.001	Referent	< 0.001
Fetal liver	22 (4.4, 43)*		− 56 (− 61, − 49)*	
Placenta	− 50 (− 57, − 42)*		− 63 (− 68, − 57)*	
**BDE-100**				
Maternal serum	Referent	< 0.001	Referent	< 0.001
Fetal liver	12 (− 6.3, 35)		− 59 (− 65, − 51)*	
Placenta	− 57 (− 64, − 49)*		− 68 (− 73, − 62)*	
**BDE-153**				
Maternal serum	Referent	< 0.001	Referent	< 0.001
Fetal liver	25 (4.1, 50)*		− 55 (− 62, − 47)*	
Placenta	− 51 (− 59, − 41)*		− 64 (− 70, − 57)*	
**ΣPBDE4**^b^				
Maternal serum	Referent	< 0.001	Referent	< 0.001
Fetal liver	27 (7.2, 50)*		− 55 (− 61, − 47)*	
Placenta	− 56 (− 63, − 48)*		− 66 (− 71, − 60)*	

*CI* confidence interval.

*p < 0.01.

^a^Calculated from censored linear regression models using maximum likelihood estimation (assuming a log-normal distribution) to account for PBDE values below the method detection limit. Wet-weight levels expressed in ng/ml (maternal serum) or ng/g (placenta and fetal liver).

^b^ΣPBDE4 includes congeners with > 50% detection frequency in all tissues (BDE-47, -99, -100, and -153).

**Figure 3:**
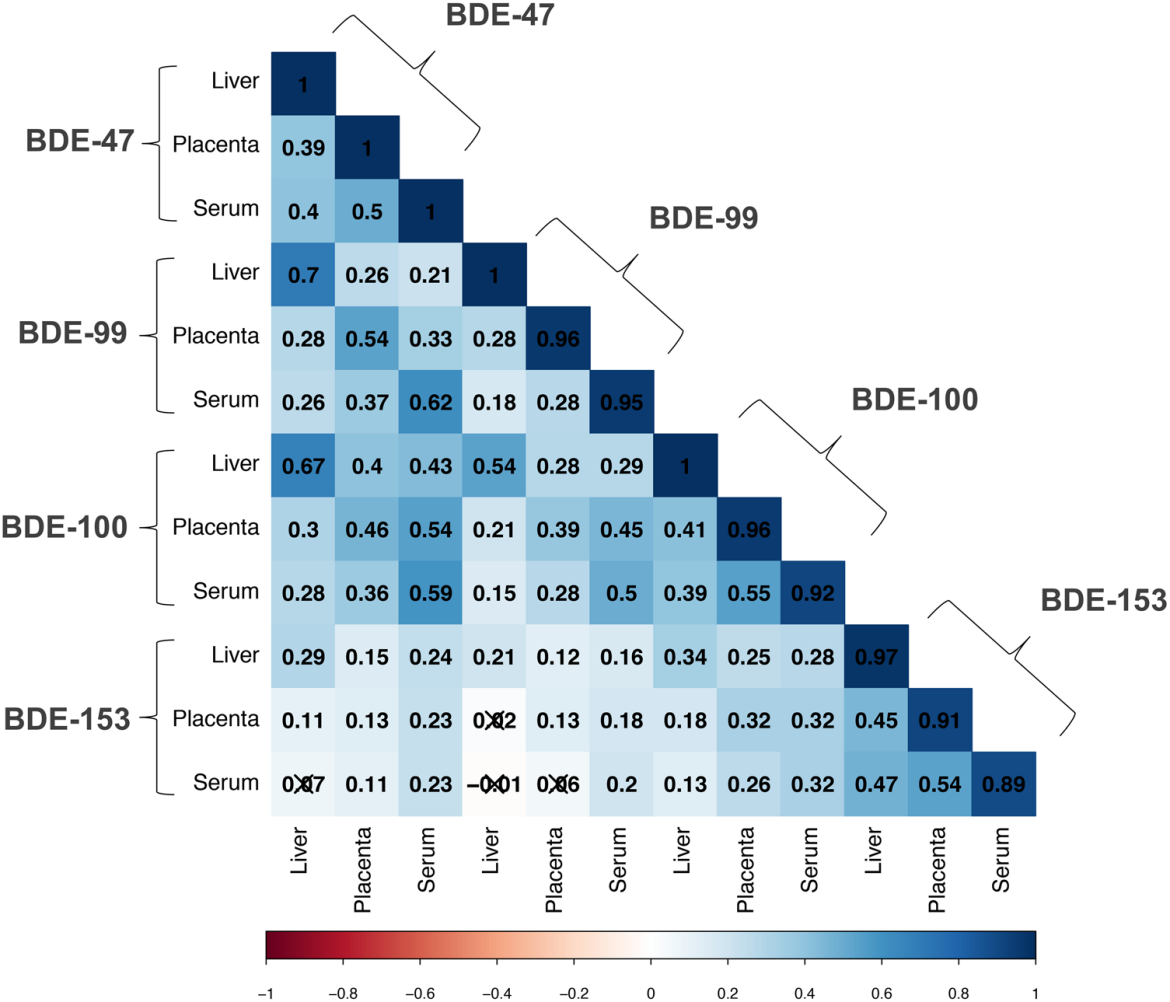
Correlation of wet-weight PBDE levels across biological matrices during midgestation (study waves 3 and 4; 2014–16; n = 180). Values and shading denote the magnitude and direction of censored Kendall’s Tau Correlation Coefficients, with null correlation values crossed out (*p* > 0.05). PBDE congeners with > 50% detection frequencies in all biological matrices were included in correlation analysis (BDE-47, -99, -100, and -153).

In general, PBDE congeners were moderately correlated across biological matrices, with higher correlations observed between maternal serum and placental tissues than between fetal liver and maternal serum or between fetal liver and placenta (Fig. 3). The least correlated congener across biological matrices was BDE-99, followed by BDE-47, BDE-153, and BDE-100. Wet-weight correlations ranged from 0.18 between fetal liver and maternal serum BDE-99 levels to 0.55 between placenta and serum BDE-100 wet-weight levels (*p* < 0.0001). A similar correlation pattern was observed for lipid-normalized values. While BDE-47, -99, and -100 were relatively correlated with each other, both within and between tissues (*τ* ≥ 0.39; *p* < 0.05), BDE-153 was the least correlated with other congeners, either within or between tissues (*τ* ≤ 0.39; *p* < 0.10) (Fig. 3).

**Figure 4:**
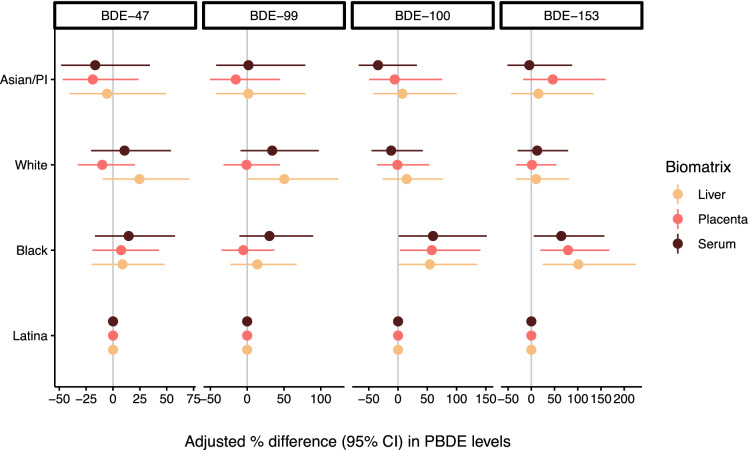
Adjusted percent (%) difference in PBDE levels between racial/ethnic subgroups by congener and biomatrix among US-born participants during midgestation (study waves 3–4; 2014–16; n = 105). Modeled differences were adjusted for educational attainment, gestational age (continuous), year of sample collection, and tissuespecific total lipid levels. Number (%) evaluated in each racial/ethnic subgroup = 37 (35%), 32 (30%), 27 (26%), and 9 (8.6%) for Latina/Hispanic, Non-Hispanic Black, Non-Hispanic White, and Asian/Pacific Islander (including Other/Native American), respectively.

We found evidence of racial/ethnic PBDE exposure disparities in maternal and fetal tissues (Non-Hispanic Black > Latina/Hispanic > Non-Hispanic White > Asian/Pacific Islander/Other; *p* < 0.001) from multivariable censored linear regression models assessing PBDE levels in each biological tissue across racial/ethnic subgroups (restricted to US-born only participants and adjusted for educational attainment, gestational weeks, year of sample collection, and total lipid levels) (Fig. 4 and Table S4). More specifically, non-Hispanic Black women had disproportionately higher PBDE levels compared to other racial/ethnic subgroups (for congeners BDE-100 and -153). We found 54% (95% CI:1.3–135%, *p* = 0.043), 58% (95% CI: 3.2–141%, *p* = 0.035), and 60% (95% CI: 1.4–151%, *p* = 0.043) increased fetal liver, placental, and maternal serum BDE-100 levels, respectively, as well as 101% (95% CI: 25–225%, *p* = 0.004), 79% (95% CI: 20–168, *p* = 0.005) and 65% (95% CI: 5.3–157, *p* = 0.029) increased fetal liver, placental, and maternal serum BDE-153 levels, respectively, among non-Hispanic Black women compared to the referent group (Latina/Hispanic women). While other racial/ethnic differences were also observed in this analysis, the differences between non-Hispanic Black and Latina/Hispanic women were the most consistent and pronounced across unadjusted and adjusted models (Fig. 4 and Table S4).

**Figure 5:**
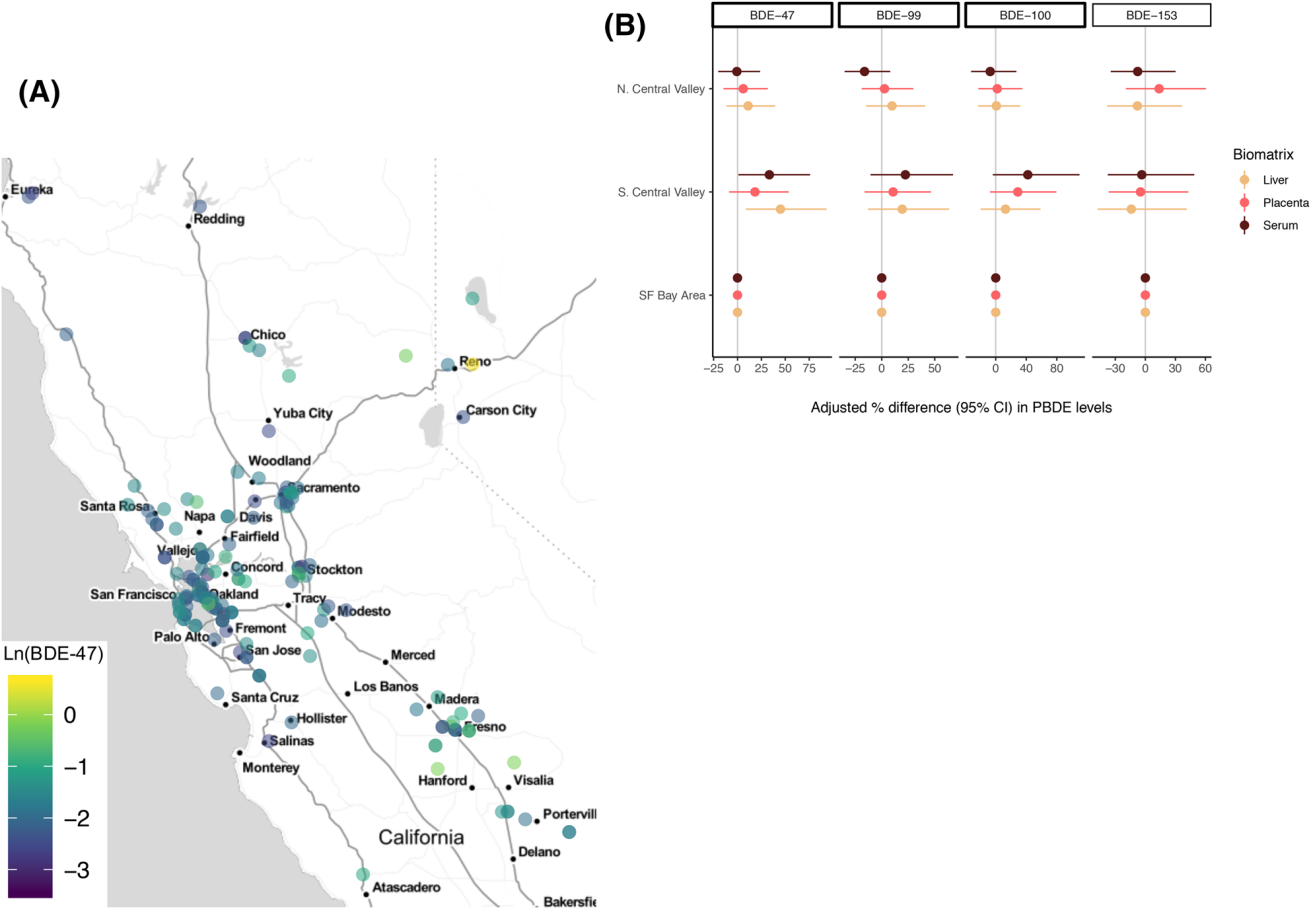
Unadjusted wet-weight BDE-47 levels in maternal serum (ng/ml) by zip code **(A)** and adjusted percent (%) difference in serum PBDE levels between geographical regions of residence by congener and biomatrix **(B)** during mid-gestation (study waves 3–4; 2014–16; n = 180). Modeled differences were adjusted for educational attainment, gestational age (continuous), year of sample collection, and tissue-specific total lipid levels as independent covariates. Number (%) evaluated in each region of residence = 99 (61%), 24 (15%), 39 (24%), for SF Bay Area, South Central Valley/Fresno, and North Central Valley (including the North Coast and Sierras), respectively. Map generated in R (Version 3.5.1) using the qmplot function (https://journal.r-project.org/archive/2013-1/kahle-wickham.pdf).

PBDE levels varied somewhat across geographical regions of residence, with study participants living in Fresno/South Central Valley having the highest serum BDE-47 levels, followed by residents of the San Francisco Bay Area and the North Central Valley (including the North Coast and Sierras) (*p* < 0.10) (Fig. 5 and Table S5). However, geographic/regional differences were attenuated with lipid adjustment and the inclusion of additional covariates in multivariable models. For example, in unadjusted models, participants living in Fresno/South Central Valley had 53% (95% CI: 14–105%, *p* = 0.01) higher serum wet-weight BDE-100 levels than SF Bay Area residents, which decreased to 41% (95% CI: 4.6–91%, *p* < 0.001) in the adjusted model. However, the association was not observed when restricting the study population to US-born only participants (Fig. 5 and Table S5).

## Discussion

This is the largest cross-sectional study to our knowledge to examine PBDE levels in matched samples of maternal serum, placenta, and fetal liver in a diverse population of pregnant women during mid-gestation (*n* = 180). We found that PBDE levels were reasonably correlated across biological compartments. Four PBDE congeners (BDE-47, 99, 100, and 153) were detected in > 50% of all biological tissues. Fetal levels were similar to or higher than maternal serum, while placental levels were on average lower. Additionally, suggestive evidence of racial/ethnic and geographic differences was observed, indicating that black women and women living in Fresno/South Central Valley may be disproportionately exposed to higher PBDE levels in maternal and fetal tissues than other sociodemographic groups, although further research is warranted.

Serum PBDE levels among pregnant women in our study population (sampled in 2014–16) were similar to or slightly lower than those reported previously among an older population of female teachers in California (sampled in 2011–15)^46^. In contrast, placental PBDE levels in this study (sampled in 2014–16) were more than two-fold higher than levels reported in another U.S. population (i.e., ~ 11 ng/g lipid compared to ~ 5 ng/g lipid^23^); however, the prior study collected samples at delivery among women in North Carolina and thus may not be directly comparable. These results are consistent with other research demonstrating disproportionate PBDE exposure burdens among Californians (compared to other North Americans and the rest of the world), which may be attributable to PBDE source differences related to California’s unique historically strict flammability laws (i.e., TB117)^7^. Moreover, we observed higher detection frequencies for all pentaBDE congeners except BDE-153 (99–100% compared to 69–91%), and a lower detection frequency for placental BDE-209 (11% in our study compared to > 50% reported previously^23^), further supporting the notion that PBDE exposure sources differ between California and other states. The lower detection of BDE-209 is also consistent with scientific literature demonstrating decreased BDE-209 levels and detection following the 2013 U.S. phase-out of decaBDE^8,10,33,47^, a widely used commercial mixture in electronics and hard plastic casings^6^. However, it is worth noting that the fully brominated BDE-209 can degrade into lower brominated congeners^48–50^ and is more challenging to measure analytically, which may in part explain its relatively lower detection. Future work to resolve these analytic challenges could help ascertain whether the widespread historic use of BDE-209 in decaBDE-treated products has resulted in higher exposures despite decreasing temporal trends.

In contrast, maternal–fetal PBDE levels in this study were comparable (though slightly lower) to our previous investigation in which placental PBDE levels were measured among a pilot sample of our study population in 2014 (*n* = 50 matched maternal, placental, and fetal samples)^21^. Interestingly, while previous research has demonstrated efficient transplacental transfer for lower-brominated PBDE congeners, the ratios of wet-weight PBDE levels between maternal and fetal liver tissues observed in this study (> 1.0) indicate the possibility of efficient transfer (leading to higher relative fetal PBDE exposures) for all PBDE congeners^28,51,52^. However, further research examining the influence of lipid adjustment on cross-tissue relationships is warranted, since maternal:fetal PBDE level ratios were altered with lipid-normalized values.

While PBDE congeners in this study were less correlated across maternal–fetal tissues than reported in a previous study wave^21^ (e.g., 0.44–0.50 in this study compared to 0.61–0.78 reported previously for BDE-47), the differential findings could be due to decreasing (and shifting) temporal trends in U.S. PBDE exposure levels following the mid-2000 pentaBDE phase-out, which increased the use of alternative flame retardants (e.g., organophosphate flame retardants) in product treatment reformulations^5^. Some researchers have hypothesized that PBDE exposure trends will follow the same pattern previously observed for polychlorinated biphenyls (PCBs)^46,53,54^. PCB exposures initially declined after their phase-out in the 1970s but eventually plateaued as the dominant route of PCB exposure was thought to shift from indoor air/dust to diet (largely as a result of increased landfill disposal of PCB-contaminated products and subsequent groundwater contamination)^53–55^. Perhaps a more likely explanation for the cross-tissue correlation differences between study waves is that we have a more robust sample size (*n* = 180) than was assessed previously (*n* = 50); however, the correlation coefficient confidence intervals were not previously reported, precluding our ability to determine whether the current estimates were within previously observed ranges. The correlation discrepancies may also be due to differences in analytical methodology between study waves. For example, the previous study wave included only participants with detectable PBDE levels in each biological matrix to calculate a Spearman’s correlation coefficient, while our censored Kendall’s tau method was able to account for values below the detection limit^21^.

Our findings indicate that specific ethnic/racial subgroups may be disproportionately exposed to PBDEs. We found that pregnant non-Hispanic black women had disproportionately higher PBDE levels (specifically congeners BDE-100 and -153) during mid-gestation than other racial/ethnic groups. Results from the multivariable analysis of PBDE levels with race/ethnicity, country of origin, and other SES variables (i.e., educational attainment) were consistent with previous data reporting higher PBDE levels among study participants who are non-white, U.S. born, and/or have lower SES^7,14,16,18–20,29,45,56^. Additionally, our findings are consistent with a previous study reporting higher cord serum PBDE levels among African American infants born in New York City between 1998 and 2006^14^. The fact that racial/ethnic PBDE level differences were based on congener type in this study may indicate differential exposure sources or pathways among racial/ethnic subgroups. For example, while diet is considered to be a dominant exposure source of BDE-153 (i.e., fish intake), dust is more highly correlated with exposure to BDE-47, -99, and -100^9,13,20,53^, which together comprised the main components of the pentaBDE flame retardant mixture used for nearly three decades in furniture and baby products sold in the United States^5^. Future investigation of PBDE exposures among black women should consider potential unique dietary exposure sources among this subpopulation.

While prior research has demonstrated an association between higher serum and dust PBDE levels with lower SES (e.g., low-income households)^7,16–20,57,58^, we did not collect information on income status in this study and therefore cannot determine whether racial/ethnic differences in the study population could have been explained by income disparities. However, we do have information on insurance status and the majority of this racially/ethnically diverse study population relied on public rather than private health insurance, indicating a lower income/SES status for the study population as a whole. Nevertheless, racial/ethnic differences persisted after adjustment for other SES variables (i.e., educational attainment), indicating the reported PBDE exposure disparities may in fact be due to exposure source differences among racial/ethnic rather than SES subgroups. Future efforts to delineate differential sources of PBDE exposure among racial/ethnic subgroups and to distinguish these from other income/SES factors would help pinpoint which sociodemographic groups are disproportionately exposed and could help further identify strategic opportunities for exposure reduction among these vulnerable groups.

This study also revealed potential geographic differences in PBDE exposure. Although the geographical variation in PBDE levels in this study were only marginally significant, women living in Fresno/South Central Valley had substantially higher levels of certain PBDEs than women residing in the San Francisco Bay Area (e.g., ~ 30% higher wet-weight BDE-47 levels; *p* = 0.07). This finding is consistent with at least one previous study which found higher PBDE levels associated with the Fresno/South Central Valley region^46^, signaling potential regional differences in PBDE exposure sources and/or routes of exposure that might warrant further study (e.g., decreased neighborhood or community SES factors, increased exposure to air pollution and/or other stressors, and/or unique attributes of agricultural land use in the Southern Central Valley). However, the lack of association observed when the analysis was restricted to US-born only participants indicates birth country may explain the observed geographic association among the pooled study population. Nevertheless, potential regional PBDE exposure disparities should be explored more comprehensively in future research on PBDE exposures among pregnant populations in California, with particular attention on Fresno and the Central Valley.

## Conclusions

Our analysis represents the largest study at present to examine matched PBDE measurements across maternal, placental, and fetal tissues during mid-gestation of pregnancy among a diverse and vulnerable population of women in Northern California and the Central Valley. We conclude that PBDEs are widely detected and differentially distributed across the maternal-placental-fetal unit. Further research is needed to identify potential modifiable sources which may contribute to disproportionate PBDE exposure burdens and their associated health risks among particular subpopulations of pregnant women in California.

## Supplementary information

Supplementary Information.

## Data Availability

The datasets used and/or analyzed during the current study are available from the corresponding author on reasonable request.

## Abbreviations

BMI: Body mass index
MDL: Method detection limit
MLE: Maximum likelihood estimation
MTBE: Methyl *tert*-butyl ether
PBDE: Polybrominated diphenyl ethers
SES: Socioeconomic status
UCSF: University of California, San Francisco
US: United States
WOC: Women’s Options Center
