# Supplementary material for: Racial/ethnic and geographic differences in polybrominated diphenyl ether (PBDE) levels across maternal, placental, and fetal tissues during mid-gestation
Source: Sci Rep. 2020 Jul 22;10:12247. doi: 10.1038/s41598-020-69067-y (PMC7376153; doi:10.1038/s41598-020-69067-y)
Supplement: Supplementary file 1 — Supplementary Information. [file 41598_2020_69067_MOESM1_ESM.pdf]

Supplemental Tables

Racial/Ethnic and Geographic Differences in Polybrominated Diphenyl Ether (PBDE) Levels across Maternal, Placental, and Fetal Tissues during Mid-gestation

*Julia R. Varshavsky[1], Saunak Sen[2], Joshua F. Robinson[1,3], Sabrina Crispo Smith[4], Julie Frankenfield[4], Yunzhu Wang[4], Greg Yeh[4], June-Soo Park[4], Susan J. Fisher[3], Tracey J. Woodruff[1]**

[1] Program on Reproductive Health and the Environment, Department of Obstetrics, Gynecology & Reproductive Sciences, University of California, San Francisco, 550 16th Street, 7th Floor, San Francisco, CA, 94143, USA

[2] University of Tennessee Health Science Center, Department of Preventive Medicine, 66 North Pauline St, Memphis, TN, 38163, USA

[3] Center for Reproductive Sciences and Department of Obstetrics, Gynecology & Reproductive Sciences, University of California, San Francisco, 513 Parnassus Avenue, San Francisco, CA, 94143, USA

[4] California Environmental Protection Agency, Department of Toxic Substances Control, Environmental Chemistry Laboratory, 700 Heinz Ave # 200, Berkeley, CA, 94710, USA

*Corresponding author:

Tracey J. Woodruff, PhD, MPH
University of California, Berkeley, School of Public Health
Program on Reproductive Health and the Environment
University of California, San Francisco
Mailstop 0132, 550 16th Street, 7th Floor
San Francisco, CA 94143
Emails: tracey.woodruff@ucsf.edu
Phone: 415-476-3197

TABLE S1. Detection frequencies for 19 PBDEs in matched samples of fetal liver, placenta, and maternal serum during mid-gestation (study waves 3–4; 2014–16; n=180).

		**Fetal Liver**				**Placenta**				**Maternal Serum**			
Congener		*MDL (ng/g)*	*Non-report†*	*N > MDL*	*% > MDL*	*MDL (ng/g)*	*Non-report†*	*N > MDL*	*% > MDL*	*MDL (ng/ml)*	*Non-report†*	*N > MDL*	*% > MDL*
Wave 3 (2014; n = 50)	BDE-17	0.008	0	2	4	0.008	0	0	0	0.008	0	0	0
	BDE-28	0.008	0	43	86	0.008	0	14	28	0.008	0	39	78
	BDE-47	0.032	0	50	100	0.032	0	48	96	0.032	0	48	96
	BDE-66	0.008	0	1	2	0.008	0	0	0	0.008	0	0	0
	BDE-85	0.008	0	3	6	0.008	0	0	0	0.008	0	2	4
	BDE-99	0.0175	0	46	92	0.018	0	30	60	0.0175	0	32	64
	BDE-100	0.008	0	46	92	0.008	0	23	46	0.008	0	32	64
	BDE-153	0.016	0	36	72	0.016	0	15	30	0.016	0	31	62
	BDE-154	0.016	0	3	6	0.016	0	1	2	0.016	0	1	2
	BDE-183	0.016	0	2	4	0.016	0	0	0	0.016	0	0	0
	BDE-196	0.016	0	0	0	0.016	0	1	2	0.091	0	1	2
	BDE-197	0.016	0	7	14	0.016	0	1	2	0.115	0	0	0
	BDE-201	0.016	0	0	0	0.016	0	0	0	0.137	0	0	0
	BDE-202	0.016	0	0	0	0.016	0	0	0	0.064	0	1	2
	BDE-203	0.016	0	0	0	0.016	0	0	0	0.032	0	5	10
	BDE-206	0.02	0	0	0	0.02	0	0	0	0.435	0	0	0
	BDE-207	0.02	0	6	12	0.02	0	1	2	0.623	0	0	0
	BDE-208	0.02	0	1	2	0.02	0	0	0	0.348	0	1	2
	BDE-209	0.15	0	3	6	0.15	0	2	4	0.864	0	1	2
Wave 4 (2014–16; n = 130)	BDE-17	0.008	0	2	1.5	0.004	1	0	0	0.02	0	0	0
	BDE-28	0.008	0	86	66	0.006	1	82	64	0.02	0	21	16
	BDE-47	0.042	0	128	99	0.017	0	130	100	0.04	0	128	99
	BDE-66	0.008	0	2	1.5	0.004	0	0	0	0.02	0	0	0
	BDE-85	0.008	0	10	7.7	0.004	0	4	3.1	0.02	0	2	1.5
	BDE-99	0.018	0	125	96	0.009	0	129	99	0.03	0	111	85
	BDE-100	0.009	0	123	95	0.004	0	130	100	0.02	0	101	78
	BDE-153	0.016	0	114	88	0.009	0	120	92	0.03	0	93	72
	BDE-154	0.016	0	4	3.1	0.008	0	5	3.8	0.03	0	0	0
	BDE-183	0.016	0	0	0	0.008	0	4	3.1	0.03	0	1	0.8
	BDE-196	0.016	1	0	0	0.008	0	2	1.5	0.03	0	0	0
	BDE-197	0.016	1	12	9.3	0.008	0	11	8.5	0.03	0	2	1.5
	BDE-201	0.016	1	0	0	0.008	0	0	0	0.03	0	0	0
	BDE-202	0.016	1	0	0	0.008	1	0	0	0.03	0	0	0
	BDE-203	0.016	1	0	0	0.008	0	2	1.5	0.03	0	0	0
	BDE-206	0.021	12	0	0	0.013	2	2	1.6	0.04	1	0	0
	BDE-207	0.021	12	10	8.5	0.014	2	11	8.6	0.04	1	1	0.8
	BDE-208	0.021	12	1	0.8	0.016	2	2	1.6	0.04	1	0	0
	BDE-209	0.200	38	8	8.7	0.066	45	9	11	0.10	13	5	4.3

MDL = Method Detection Limit. Green color indicates > 50% detection frequency.

† Not reported due to sample loss or surrogate failure. Not included in detection frequency calculation.

TABLE S2. Unadjusted and adjusted associations between wet-weight and lipid-adjusted PBDE levels in paired maternal serum and fetal liver samples[a] during mid-gestation (study waves 2–4; n=224)

	Wet-weight (ng/g)				***Lipid-adjusted (ng/g lipid)***			
**Model**	**Beta**	**SE**	**p-value**	**adjusted R2**	**Beta**	**SE**	**p-value**	**adjusted R2**
**BDE-47**								
unadjusted	0.76	0.08	0	0.36	0.76	0.05	0	0.53
adjusted	0.76	0.08	0	0.36	0.76	0.05	0	0.53
**BDE-99**								
unadjusted	0.40	0.09	0	0.09	0.43	0.07	0	0.16
adjusted	0.51	0.09	0	0.21	0.51	0.07	0	0.21
**BDE-100**								
unadjusted	0.55	0.07	0	0.27	0.6	0.05	0	0.41
adjusted	0.74	0.06	0	0.46	0.73	0.05	0	0.52
**BDE-153**								
unadjusted	0.80	0.07	0	0.41	0.86	0.06	0	0.51
adjusted	0.88	0.07	0	0.44	0.89	0.06	0	0.55

[a] Unadjusted and adjusted associations between maternal serum (independent exposure; wet-weigh,; *ng/ml*) and fetal liver (dependent outcome; wet-weight, *ng/g;* and lipid-adjusted; *ng/g lipid*) ln-PBDE levels were evaluated using linear regression models, where PBDE levels below method detection limit (MDL) were substituted with MDL/sqrt(2). Adjusted models included gestational age, fetal sex, study wave, and total serum lipid levels as additional explanatory variables.

TABLE S3. Descriptive statistics on tissue-specific total lipid levels in matched samples of maternal, placental, and fetal liver tissues (n=180; 2014–16) and on maternal blood pressure among the whole study population (study waves 1–4; N=249; 2008–16) during mid-gestation.

**Total Lipids**[a]	**n**	**Mean**	**Median**	**IQR**	**Min**	**Max**
Fetal Liver (mg/g)	180	22	23	9.0	4.9	39
Placental (mg/g)	180	10	10	3.1	5.2	18
Maternal Serum (mg/ml)	180	7.0	6.9	1.5	4.5	11

IQR = Interquartile range (25th–75th percentile).
[a]Tissue-specific total lipid levels expressed as *mg/ml* in maternal serum and *mg/g* in placental and fetal liver tissues.

TABLE S4. Unadjusted and adjusted percent (%) difference in PBDE levels† by race/ethnicity during mid-gestation (study waves 3–4; 2014–16; n=180).

			**Model 1 (unadjusted)**		**Model 2 (adjusted)**		**Model 3 (adjusted US-born)**	
Biomatrix	***Congener***	***Race/ ethnicity***	***% Difference (95% CI)***	***p-value***	***% Difference (95% CI)***	***p-value***	***% Difference (95% CI)***	***p-value***
Fetal Liver	BDE-47	*Latina*	Referent	0.060	Referent	<0.001	Referent	<0.001
		Black	-6.3 (-32, 30)		8.3 (-16, 39)		8.8 (-20, 48)	
		White	20 (-14, 69)		44 (9.9, 88)***		25 (-9.2, 71)	
		Asian/PI	-28 (-51, 6.9)		-18 (-40, 13)		-5.6 (-40, 49)	
	BDE-99	*Latina*	Referent	0.100	Referent	<0.001	Referent	<0.001
		Black	-0.3 (-30, 42)		15 (-12, 51)		14 (-23, 67)	
		White	37 (-5.5, 97)*		58 (18, 111)***		50 (1.3, 123)**	
		Asian/PI	-8.0 (-40, 41)		0.9 (-28, 41)		1.5 (-43, 79)	
	BDE-100	*Latina*	Referent	0.080	Referent	<0.001	Referent	<0.001
		Black	29 (-10, 85)		50 (11, 103)***		54 (1.3, 135)**	
		White	16 (-21, 70)		41 (1.7, 94)**		15 (-26, 77)	
		Asian/PI	-22 (-50, 21)		-3.4 (-34, 41)		7.3 (-42, 100)	
	BDE-153	*Latina*	Referent	0.002	Referent	<0.001	Referent	<0.001
		Black	41 (-10, 121)		88 (25, 183)***		101 (25, 225)***	
		White	19 (-26, 92)		41 (-9.0, 117)		10 (-33, 81)	
		Asian/PI	-50 (-72, -11)**		-38 (-63, 3.7)*		15 (-43, 134)	
Placenta	BDE-47	*Latina*	Referent	0.100	Referent	<0.001	Referent	<0.001
		Black	3.1 (-19, 30)		7.8 (-14, 35)		7.5 (-19, 43)	
		White	0.7 (-21, 29)		12 (-12, 42)		-10 (-33, 20)	
		Asian/PI	-24 (-43, 1.2)*		-13 (-34, 15)		-19 (-47, 24)	
	BDE-99	*Latina*	Referent	0.300	Referent	<0.001	Referent	0.010
		Black	-8.8 (-29, 17)		-4.3 (-25, 23)		-5.3 (-34, 37)	
		White	-3.8 (-26, 25)		8.8 (-16, 42)		-0.9 (-32, 45)	
		Asian/PI	-20 (-41, 8.0)		-10 (-34, 22)		-16 (-51, 44)	
	BDE-100	*Latina*	Referent	0.100	Referent	<0.001	Referent	<0.001
		Black	18 (-13, 59)		37 (2.1, 85)**		58 (3.2, 141)**	
		White	-4.0 (-30, 32)		11 (-19, 52)		-1.0 (-36, 53)	
		Asian/PI	-22 (-46, 13)		-7.9 (-36, 33)		-5.7 (-49, 75)	
	BDE-153	*Latina*	Referent	0.010	Referent	<0.001	Referent	<0.001
		Black	59 (11, 127)**		86 (32, 162)***		79 (20, 168)***	
		White	8.8 (-25, 59)		21 (-16, 74)		1.4 (-33, 54)	
		Asian/PI	-13 (-44, 35)		-1.9 (-36, 50)		46 (-18, 160)	
Maternal Serum	BDE-47	*Latina*	Referent	0.020	Referent	<0.001	Referent	0.010
		Black	-3.4 (-25, 24)		7.7 (-17, 39)		15 (-17, 58)	
		White	3.5 (-21, 35)		18 (-9.6, 55)		11 (-20, 54)	
		Asian/PI	-33 (-51, -8.9)**		-21 (-42, 8.6)		-17 (-48, 34)	
	BDE-99	*Latina*	Referent	0.100	Referent	<0.001	Referent	0.010
		Black	3.4 (-23, 39)		20 (-11, 61)		30 (-11, 90)	
		White	10 (-19, 50)		25 (-8.5, 70)		34 (-9.0, 97)	
		Asian/PI	-26 (-48, 6.7)		-13 (-40, 25)		1.7 (-42, 79)	
	BDE-100	*Latina*	Referent	0.010	Referent	<0.001	Referent	<0.001
		Black	26 (-15, 86)		64 (13, 138)***		60 (1.4, 151)**	
		White	-17 (-45, 26)		8.7 (-27, 62)		-12 (-45, 42)	
		Asian/PI	-40 (-63, -2.1)**		-18 (-49, 32)		-34 (-67, 32)	
	BDE-153	*Latina*	Referent	0.001	Referent	<0.001	Referent	<0.001
		Black	62 (8.5, 142)**		99 (34, 195)***		65 (5.3, 157)**	
		White	36 (-11, 107)		60 (6.1, 143)**		13 (-29, 79)	
		Asian/PI	-38 (-63, 4.2)*		-23 (-54, 29)		-4.4 (-51, 88)	

† The % difference in PBDE levels between racial/ethnic groups estimated from censored maximum likelihood regression models, where the largest racial/ethnic subgroup was selected as the referent group (Latina/Hispanic, n=67). Model 1 was unadjusted and included all study participants with matched maternal-fetal tissues (n=180); model 2 restricted to non-missing covariate data (n=179) and adjusted for educational attainment, gestational weeks, collection year, and total lipid level (*mg/ml* in maternal serum; *mg/g* in placenta and fetal liver), while model 3 further restricted to known US-born only study participants (n=105). ***$p < 0.01$. **$p < 0.05$. *$p < 0.10$.

TABLE S5. Unadjusted and adjusted percent (%) difference in PBDE levels† by geographic region of residence during mid-gestation (study waves 3–4; 2014–16; n=180).

		Model 1 (unadjusted)		***Model 2 (adjusted)***		***Model 3 (adjusted US-born)***	
	Region of Residence	***% Diff (95% CI)***	***p-value***	***% Diff (95% CI)***	***p-value***	***% Diff (95% CI)***	***p-value***
Fetal Liver							
BDE-47	SF Bay Area	Referent	0.10	Referent	<0.001	Referent	<0.001
	S. Central Valley/Fresno	48 (0.9, 117)**		46 (8.8, 96)**		6.6 (-29, 59)	
	N. Central Valley/Coast/Sierras	22 (-12, 67)		15 (-10, 47)		-0.8 (-27, 34)	
BDE-99	SF Bay Area	Referent	0.40	Referent	<0.001	Referent	<0.001
	S. Central Valley/Fresno	23 (-20, 88)		21 (-13, 69)		3.8 (-38, 75)	
	N. Central Valley/Coast/Sierras	23 (-14, 75)		14 (-13, 51)		2.2 (-31, 51)	
BDE-100	SF Bay Area	Referent	0.80	Referent	<0.001	Referent	<0.001
	S. Central Valley/Fresno	13 (-25, 72)		12 (-21, 57)		-27 (-55, 20)	
	N. Central Valley/Coast/Sierras	4.2 (-26, 47)		0.9 (-24, 34)		-7.4 (-36, 34)	
BDE-153	SF Bay Area	Referent	0.90	Referent	<0.001	Referent	<0.001
	S. Central Valley/Fresno	7.8 (-38, 88)		-12 (-48, 48)		-34 (-66, 28)	
	N. Central Valley/Coast/Sierras	-6.9 (-41, 48)		-8.2 (-41, 42)		-22 (-52, 29)	
Placenta							
BDE-47	SF Bay Area	Referent	0.20	Referent	<0.001	Referent	<0.001
	S. Central Valley/Fresno	19 (-10, 57)		18 (-8.8, 53)		2.3 (-29, 48)	
	N. Central Valley/Coast/Sierras	11 (-12, 40)		7.0 (-14, 33)		-1.2 (-25, 31)	
BDE-99	SF Bay Area	Referent	0.05	Referent	0.09	Referent	<0.001
	S. Central Valley/Fresno	16 (-13, 56)		12 (-16, 48)		9.9 (-31, 75)	
	N. Central Valley/Coast/Sierras	7.5 (-16, 38)		4.7 (-18, 33)		2.9 (-28, 47)	
BDE-100	SF Bay Area	Referent	0.40	Referent	0.01	Referent	<0.001
	S. Central Valley/Fresno	37 (-2.7, 92)*		30 (-6.8, 82)		12 (-35, 92)	
	N. Central Valley/Coast/Sierras	-0.5 (-25, 32)		4.4 (-22, 39)		13 (-25, 71)	
BDE-153	SF Bay Area	Referent	0.03	Referent	0.20	Referent	<0.001
	S. Central Valley/Fresno	5.7 (-31, 62)		-3.6 (-36, 46)		-39 (-64, 3.0)*	
	N. Central Valley/Coast/Sierras	4.2 (-27, 50)		16 (-19, 65)		-10 (-40, 33)	
Maternal Serum							
BDE-47	SF Bay Area	Referent		Referent		Referent	<0.001
	S. Central Valley/Fresno	53 (14, 105)***	0.02	41 (4.6, 91)**	<0.001	21 (-22, 86)	
	N. Central Valley/Coast/Sierras	0.9 (-21, 29)		0.8 (-21, 29)		9.5 (-20, 50)	
BDE-99	SF Bay Area	Referent		Referent		Referent	<0.001
	S. Central Valley/Fresno	44 (3.5, 100)**	0.02	30 (-8.1, 83)	<0.001	15 (-31, 93)	
	N. Central Valley/Coast/Sierras	-15 (-36, 12)		-14 (-35, 14)		-11 (-39, 29)	
BDE-100	SF Bay Area	Referent		Referent		Referent	<0.001
	S. Central Valley/Fresno	80 (15, 180)***	0.01	55 (0.4, 141)**	<0.001	30 (-29, 140)	
	N. Central Valley/Coast/Sierras	-13 (-40, 27)		-6.7 (-35, 35)		-5.5 (-40, 49)	
BDE-153	SF Bay Area	Referent		Referent		Referent	<0.001
	S. Central Valley/Fresno	23 (-24, 99)	0.70	3.8 (-37, 70)	0.01	-26 (-60, 37)	
	N. Central Valley/Coast/Sierras	-2.2 (-35, 46)		-2.6 (-35, 46)		-21 (-50, 23)	

† The % difference in PBDE levels between geographic regions estimated from censored maximum likelihood regression models, where the largest regional subgroup was selected as the referent group (SF Bay Area, *n*=99). Model 1 was unadjusted and included all study participants with matched maternal-fetal tissues and non-missing zip codes (*n*=162); model 2 further restricted to non-missing covariate data (n=161) and adjusted for educational attainment, gestational weeks, collection year, and total lipid level (*mg/ml* in maternal serum; *mg/g* in placenta and fetal liver), while model 3 further restricted to known US-born only study participants (n=95). ***$p < 0.01$. **$p < 0.05$. *$p < 0.10$.
